# Supplementary material for: Structural basis of double-stranded RNA recognition by the J2 monoclonal antibody
Source: Nat Commun. 2025 Dec 13;17:635. doi: 10.1038/s41467-025-67414-z (PMC12816604; doi:10.1038/s41467-025-67414-z)
Supplement: Supplementary file 1 — Supplementary Information [file 41467_2025_67414_MOESM1_ESM.pdf]

**Supplementary Materials for:**

# Structural basis of double-stranded RNA recognition by the J2 monoclonal antibody

Charles Bou-Nader, Kevin M. Juma, Ankur Bothra, Andrew J. Brasington, Rodolfo Ghirlando, Motoshi Suzuki, David N. Garboczi, Stephen H. Leppla, and Jinwei Zhang

**Supplementary Table 1**
**Supplementary Figures 1-14**

| Ligand (J2 IgG) | Analyte (dsRNA) | $k_{on}$ (×$10^5$ $M^{-1}s^{-1}$) | $k_{off}$ (×$10^{-3}$ $s^{-1}$) | $K_d$ (nM, kinetics) | $K_d$ (nM, steady state) |
|---|---|---|---|---|---|
| WT | 6-bp | N.D. | N.D. | N.D. | N.D. |
| WT | 10-bp | N.D. | N.D. | N.D. | N.D. |
| WT | 14-bp | 2.3 ± 1.9 | 220.3 ± 27.8 | 1400 ± 800 | 2100 ± 600 |
| WT | 20-bp | 5.9 ± 2.8 | 72.1 ± 2.5 | 150 ± 80 | 120 ± 10 |
| WT | 30-bp | 6.7 ± 5.5 | 19 ± 15 | 37 ± 7 | 47 ± 4 |
| WT | 40-bp | 5.3 ± 0.2 | 17.2 ± 2.8 | 33 ± 6 | 80 ± 15 |
| WT | 50-bp | N.D. | N.D. | N.D. | 22 ± 8 |
| | | | | | |
| HC A30P | 20-bp | 2.1 ± 1.9 | 55.1 ± 7.0 | 28 ± 14 | 120 ± 20 |
| HC Y50A | 20-bp | N.D. | N.D. | N.D. | N.D. |
| HC Y50F | 20-bp | 15.0 ± 5.3 | 124.7 ± 29.1 | 94 ± 49 | 430 ± 40 |
| HC Y52A | 20-bp | N.D. | N.D. | N.D. | N.D. |
| HC Y52F | 20-bp | 5.8 ± 0.3 | 122.7 ± 7.1 | 210 ± 20 | 220 ± 0 |
| HC Y54A | 20-bp | 5.1 ± 0.2 | 138.0 ± 7.8 | 270 ± 10 | 300 ± 30 |
| HC Y54F | 20-bp | 5.7 ± 0.8 | 140.3 ± 6.8 | 250 ± 40 | 710 ± 170 |
| HC N55A | 20-bp | N.D. | N.D. | N.D. | N.D. |
| HC N101A | 20-bp | N.D. | N.D. | N.D. | N.D. |
| HC N101R | 20-bp | N.D. | N.D. | N.D. | N.D. |
| HC P102A | 20-bp | N.D. | N.D. | N.D. | N.D. |
| HC W104A | 20-bp | N.D. | N.D. | N.D. | N.D. |
| LC Y31A | 20-bp | 8.0 ± 3.3 | 136.8 ± 91.4 | 207 ± 21 | 940 ± 560 |
| LC Y31F | 20-bp | 7.6 ± 1.0 | 97.0 ± 3.5 | 130 ± 20 | 360 ± 60 |
| LC S33R | 20-bp | N.D. | N.D. | N.D. | N.D. |
| LC Y38F | 20-bp | 9.5 ± 0.1 | 79.0 ± 13.4 | 83 ± 14 | 150 ± 20 |
| LC W56A | 20-bp | N.D. | N.D. | N.D. | N.D. |
| LC Y101A | 20-bp | N.D. | N.D. | N.D. | N.D. |
| | | | | | |
| WT | GC_0%, 30 bp | 5.2 ± 1.9 | 146.7 ± 4.0 | 320 ± 140 | 290 ± 60 |
| WT | GC_3%, 30 bp | 1.2 ± 1.7 | 19.1 ± 24.8 | 190 ± 50 | 220 ± 90 |
| WT | GC_17%, 30 bp | 2.4 ± 0.2 | 16.4 ± 0.8 | 470 ± 30 | 36 ± 22 |
| WT | GC_33%, 30 bp | 1.4 ± 0.2 | 148.3 ± 6.7 | 1100 ± 200 | 460 ± 90 |
| WT | GC_47%, 30 bp | 1.6 ± 1.3 | 19.5 ± 2.9 | 560 ± 810 | 110 ± 20 |
| WT | GC_63%, 30 bp | N.D. | N.D. | N.D. | N.D. |
| WT | GC_77%, 30 bp | N.D. | N.D. | N.D. | N.D. |
| WT | GC_90%, 30 bp | N.D. | N.D. | N.D. | N.D. |
| WT | GC_100%, 30 bp | N.D. | N.D. | N.D. | N.D. |
| | | | | | |
| WT | Poly (rAU+rAU), 40 bp | 9.2 ± 10.6 | 103.2 ± 30.8 | 310 ± 340 | 91 ± 11 |

**Supplementary Table 1. Summary of BLI analysis parameters.** HC: heavy chain; LC: light chain. N.D.: not determined. $K_d$ (kinetics) was calculated from $k_{off}/k_{on}$ determined by kinetic analyses; $K_d$ (steady state) was derived by curve-fitting sensor responses (nm) as functions of dsRNA concentrations. See Methods for details. Values are mean ± s.d. of three biologically independent samples. All J2 used in BLI analyses are in the form of IgG.

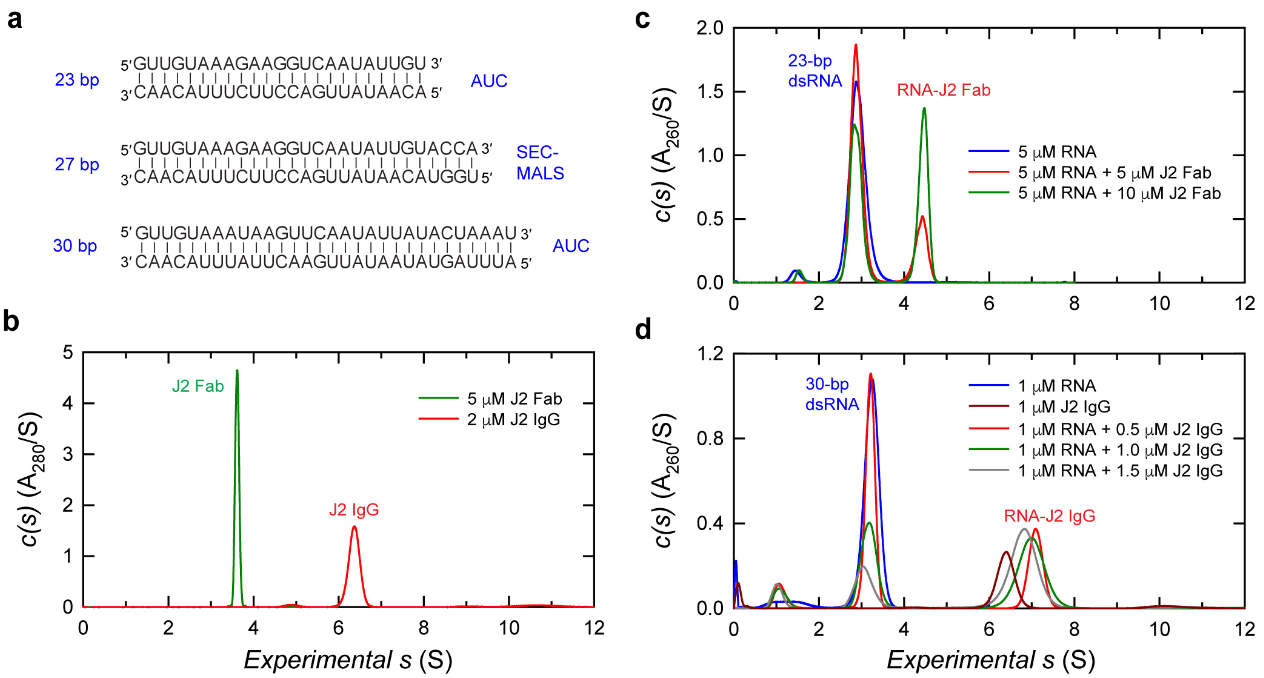


**Supplementary Figure 1. Sedimentation Velocity – Analytical Ultracentrifugation (SV-AUC) analyses of dsRNA interactions with J2 Fab and J2 IgG. a**. Sequences of dsRNAs used for AUC and SEC-MALS analyses. **b**. Absorbance c(s) distributions of 5 μM J2 Fab (green) and 2 μM J2 IgG (red). The sample of J2 Fab shows a single species at 3.61 S and 52 kDa indicative of a monomeric Fab. The sample of J2 IgG shows a species at 6.35 S and 147 kDa, indicative of a monomeric IgG. The IgG monomer accounts for 81% of the absorbing sedimenting signal; the dimer (~ 7%) and faster sedimenting material contribute to the signal. **c**. Titration of a 23-bp dsRNA (**a**) with J2 Fab (**b**). The 23-bp dsRNA shows a species at 2.90 S and 14 kDa, indicative of the expected dsRNA duplex. Addition of 1.0 (red) or 2.0 (green) molar equivalents of J2 Fab results in the contribution of a species at 4.39 S. This species sediments faster than the J2 Fab (3.61 S), indicating a 1:1 RNA:Fab complex. **d**. Titration of a 30-bp dsRNA (17% GC, **a**) with J2 IgG (**b**). The 30-bp dsRNA shows a species at 3.23 S and 19 kDa, indicative of the expected dsRNA duplex. Adding 0.5 equivalents of J2 IgG (red) results in the contribution of a species at 7.09 S. The species sediments faster than J2 IgG (6.35 S), indicating a 1:1 RNA:IgG complex. The addition of 1.0 (green) or 1.5 (gray) molar equivalents of J2 IgG shows a similar faster sedimenting species with increasing contributions from unresolved free IgG. The plot in brown shows a portion of the c(s) sedimentation profile for 1 μM free J2 IgG. Sample losses were observed due to the formation of large aggregates between the dsRNA and the IgG multimer and faster sedimenting material. Data for (**b**) were collected at 50,000 rpm and 280 nm, whereas data for (**c**) and (**d**) were collected at 50,000 rpm and 260 nm. 12 mm pathlength cells were used in (**b**) and (**d**), whereas 3 mm pathlength cells were used in (**c**).

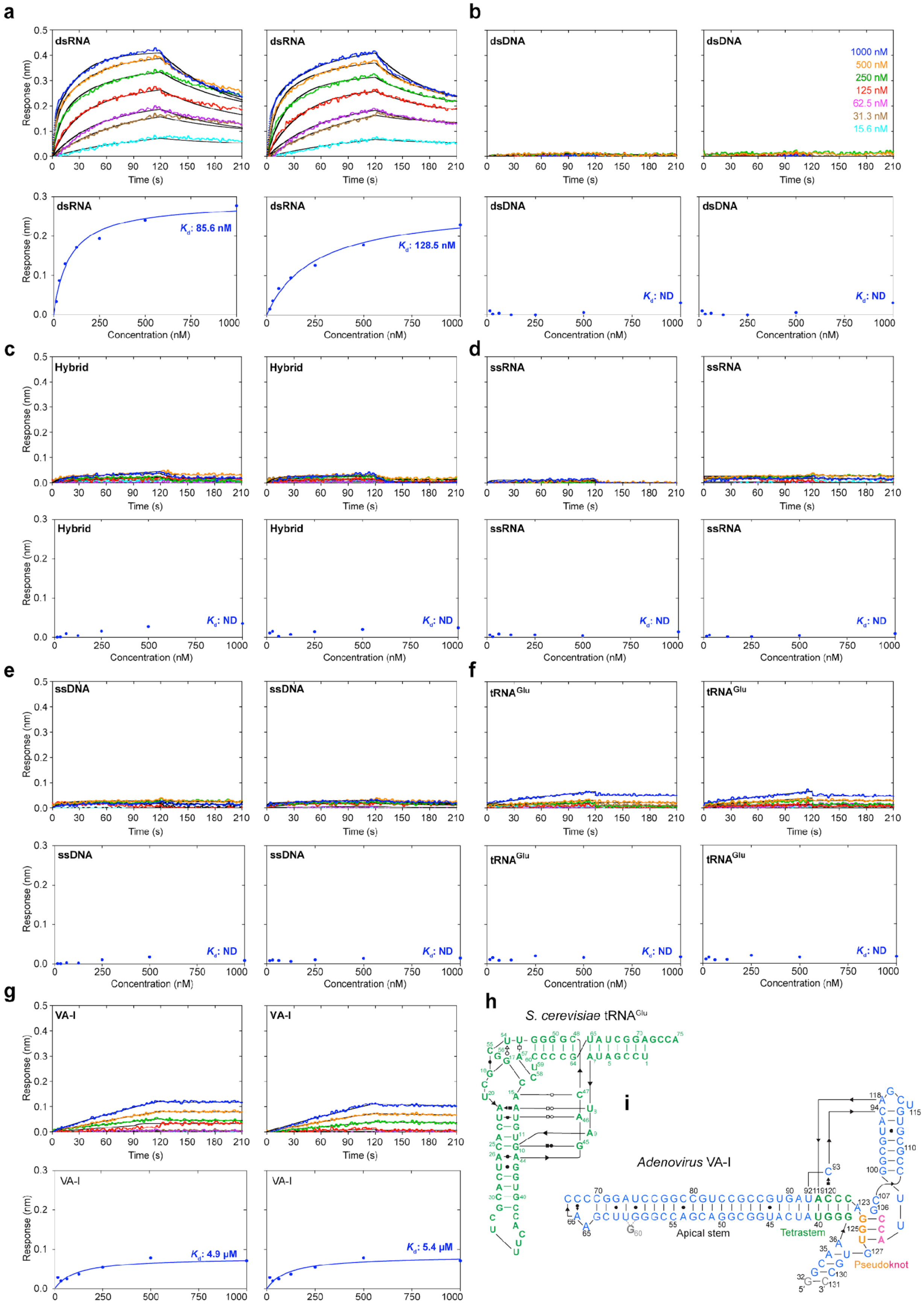
a
dsRNA
Response (nm)
Time (s)
Kd: 85.6 nM
Kd: 128.5 nM
Concentration (nM)
b
dsDNA
1000 nM
500 nM
250 nM
125 nM
62.5 nM
31.3 nM
15.6 nM
Kd: ND
c
Hybrid
d
ssRNA
e
ssDNA
f
tRNA^Glu
g
VA-I
Kd: 4.9 μM
Kd: 5.4 μM
h
S. cerevisiae tRNA^Glu
i
Adenovirus VA-I
Apical stem
Tetrastem
Pseudoknot

**Supplementary Figure 2. J2 IgG binding to different types of nucleic acids. a**-**g**, Additional BLI sensorgrams of J2 IgG binding to dsRNA (**a**), dsDNA (**b**), RNA-DNA hybrid (**c**), ssRNA (**d**), ssDNA (**e**), $tRNA^{Glu}$ (**f**), or VA-I (**g**) at 400 mM KCl. **h**. Secondary structure of $tRNA^{Glu}$ used in **f**. **i**. Secondary structure of VA-I used in **g**. Other nucleic acids used in (**a**-**f**) are shown in Fig. 1a.

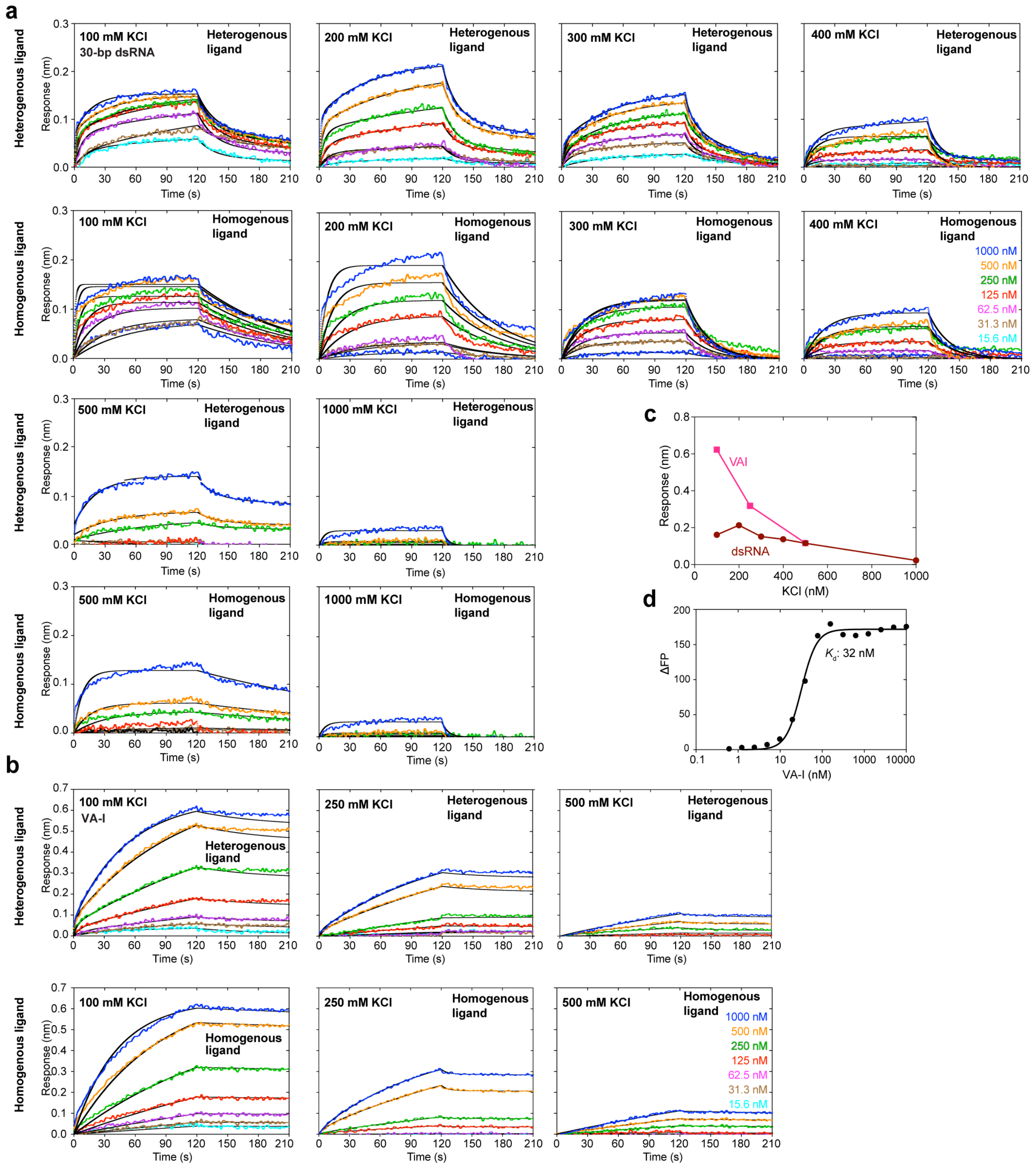


**Supplementary Figure 3. Effects of ionic strengths on J2 IgG binding to dsRNA or VA-I. a**, BLI sensorgrams of J2 IgG binding to a 30-bp dsRNA, at 100, 200, 300, 400, 500, or 1000 mM KCl, fit to the heterogeneous ligand (upper) or homogeneous ligand (lower) model. **b**. BLI sensorgrams of J2 binding to VA-I RNA at 100, 250, or 500 mM KCl, fit to the heterogeneous ligand (upper) or homogeneous ligand (lower) model . **c**, Plots of maximum sensor responses as functions of KCl concentrations, derived from **a** & **b**. **d**, Additional bimolecular fluorescence polarization (FP) analysis of J2 IgG binding to a 3′-FAM-labelled VA-I RNA. ΔFP: change in FP expressed in dimensionless millipolarization (mP) units. Experiment performed twice.

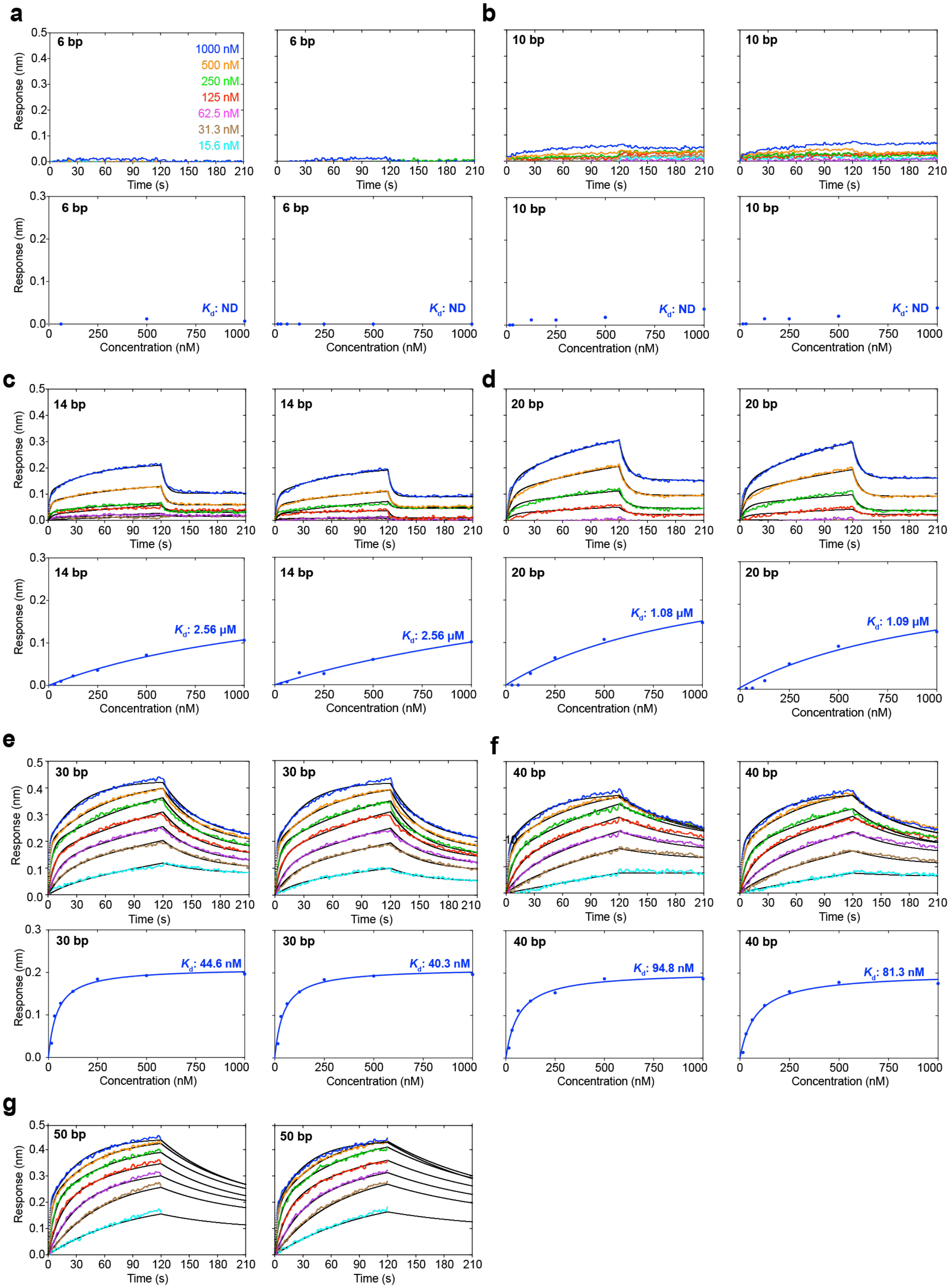
a
b
c
d
e
f
g
6 bp
10 bp
14 bp
20 bp
30 bp
40 bp
50 bp
1000 nM
500 nM
250 nM
125 nM
62.5 nM
31.3 nM
15.6 nM
Response (nm)
Time (s)
Concentration (nM)
$K_d$: ND
$K_d$: 2.56 μM
$K_d$: 1.08 μM
$K_d$: 1.09 μM
$K_d$: 44.6 nM
$K_d$: 40.3 nM
$K_d$: 94.8 nM
$K_d$: 81.3 nM

**Supplementary Figure 4. J2 IgG binding to dsRNAs of different lengths. a-g**, Additional BLI sensorgrams (upper) and steady-state analyses (lower) of J2 IgG binding to 6 (**a**), 10 (**b**), 14 (**c**), 20 (**d**), 30 (**e**), 40 (**f**), or 50 bp (**g**) dsRNAs. Sequences shown in Fig. 1e. Due to significant dependency of binding on ionic strength, 100 mM KCl was used for 6, 10, 14, and 20 bp dsRNAs; 200 mM KCl for 30 bp dsRNA; 400 mM KCl for 40 and 50 bp dsRNAs.

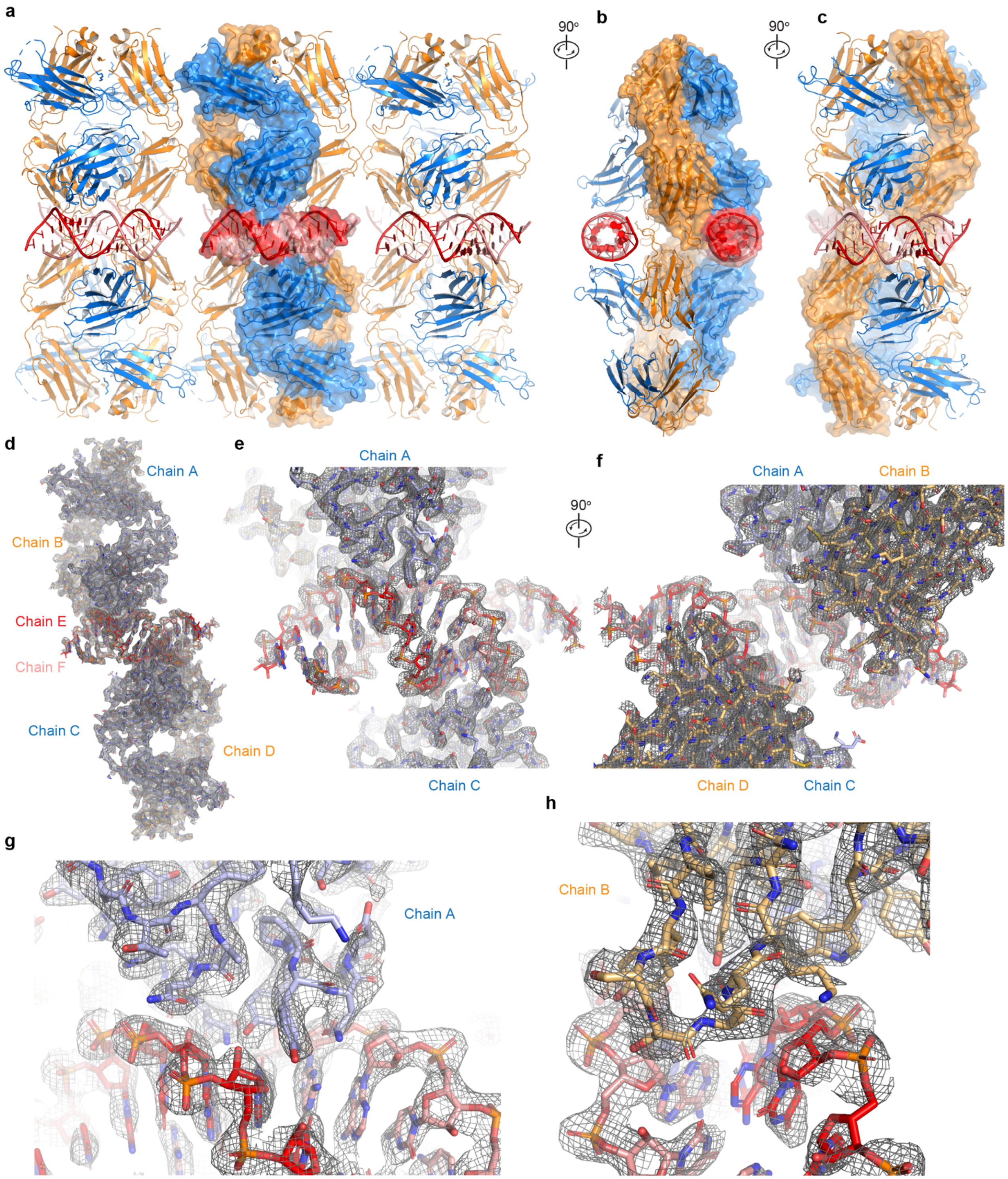
a
90°
b
90°
c
d
Chain A
Chain B
Chain E
Chain F
Chain C
Chain D
e
Chain A
Chain C
90°
f
Chain A
Chain B
Chain D
Chain C
g
Chain A
h
Chain B

**Supplementary Figure 5. Crystal packing arrangements and representative electron densities.** **a**, Crystal-packing arrangements of the J2 Fab – dsRNA molecular assemblies. The heavy and light chains of the J2 Fabs are shown in blue and orange, respectively. The two dsRNA strands are in red and salmon. Contents within a single asymmetric unit are highlighted in translucent surface representation. **b**, 90° rotated view of **a**, showing two asymmetric units. **c**, 90° rotated view of **b**, showing two asymmetric units, and a rear view relative to **a**. **d**, Composite simulated anneal-omit 2Fo-Fc density calculated using the final model of two J2 Fabs bound to a 17-bp dsRNA, superimposed with the final refined model, contoured at 0.8 σ. **e**, **f**, Two views of a portion of the map in **d** showing the J2 interface with the dsRNA strands. **g**, Portion of the map in **d** showing the J2 heavy chain interface with the dsRNA. **h**, Portion of the map in **d** showing the J2 light chain interface with the dsRNA.

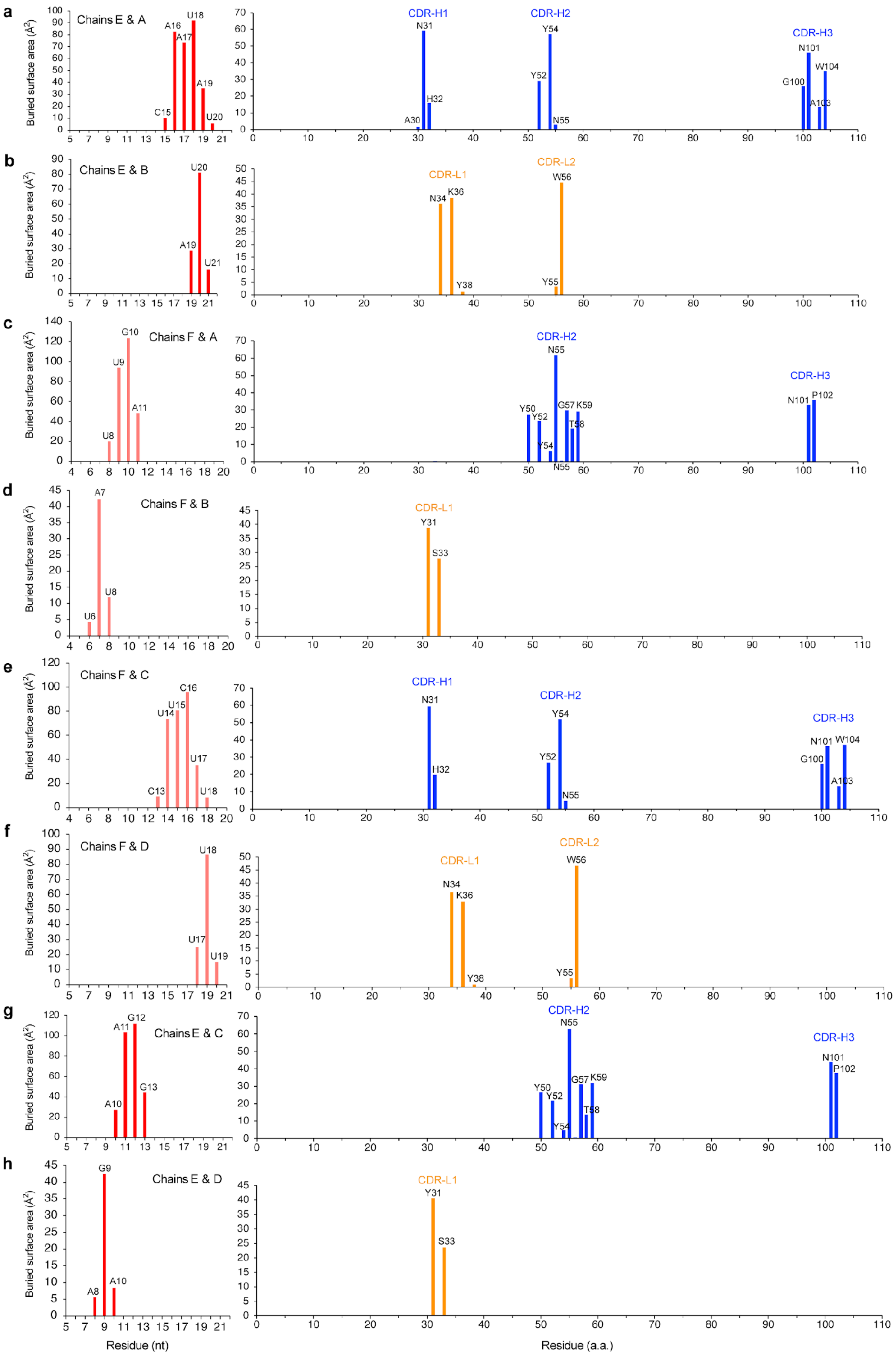

a
Chains E & A
Buried surface area (Å²)
C15
A16
A17
U18
A19
U20
CDR-H1
N31
A30
H32
CDR-H2
Y54
Y52
N55
CDR-H3
N101
W104
G100
A103
b
Chains E & B
Buried surface area (Å²)
U20
A19
U21
CDR-L1
N34
K36
Y38
CDR-L2
W56
Y55
c
Chains F & A
Buried surface area (Å²)
G10
U9
A11
U8
CDR-H2
N55
Y50
Y52
Y54
G57
K59
T58
CDR-H3
N101
P102
d
Chains F & B
Buried surface area (Å²)
A7
U8
U6
CDR-L1
Y31
S33
e
Chains F & C
Buried surface area (Å²)
C16
U14
U15
U17
C13
U18
CDR-H1
N31
H32
CDR-H2
Y54
Y52
N55
CDR-H3
N101
W104
G100
A103
f
Chains F & D
Buried surface area (Å²)
U18
U17
U19
CDR-L1
N34
K36
Y38
CDR-L2
W56
Y55
g
Chains E & C
Buried surface area (Å²)
A11
G12
A10
G13
CDR-H2
N55
Y50
Y52
Y54
G57
K59
T58
CDR-H3
N101
P102
h
Chains E & D
Buried surface area (Å²)
G9
A8
A10
CDR-L1
Y31
S33
Residue (nt)
Residue (a.a.)

**Supplementary Figure 6. Solvent-accessible surfaces buried at the J2 Fab – dsRNA interfaces.** **a**-**d**, Plots of solvent-accessible surface area (Å$^2$) buried per residue on the dsRNA strands (left, chain E or F) and the first J2 Fab (right, heavy chain A in blue; light chain B in orange). **e**-**h**, Plots of solvent-accessible surface area (Å$^2$) buried per residue on the dsRNA strands (left, chain F or E) and the second J2 Fab (right, heavy chain C in blue; light chain D in orange).

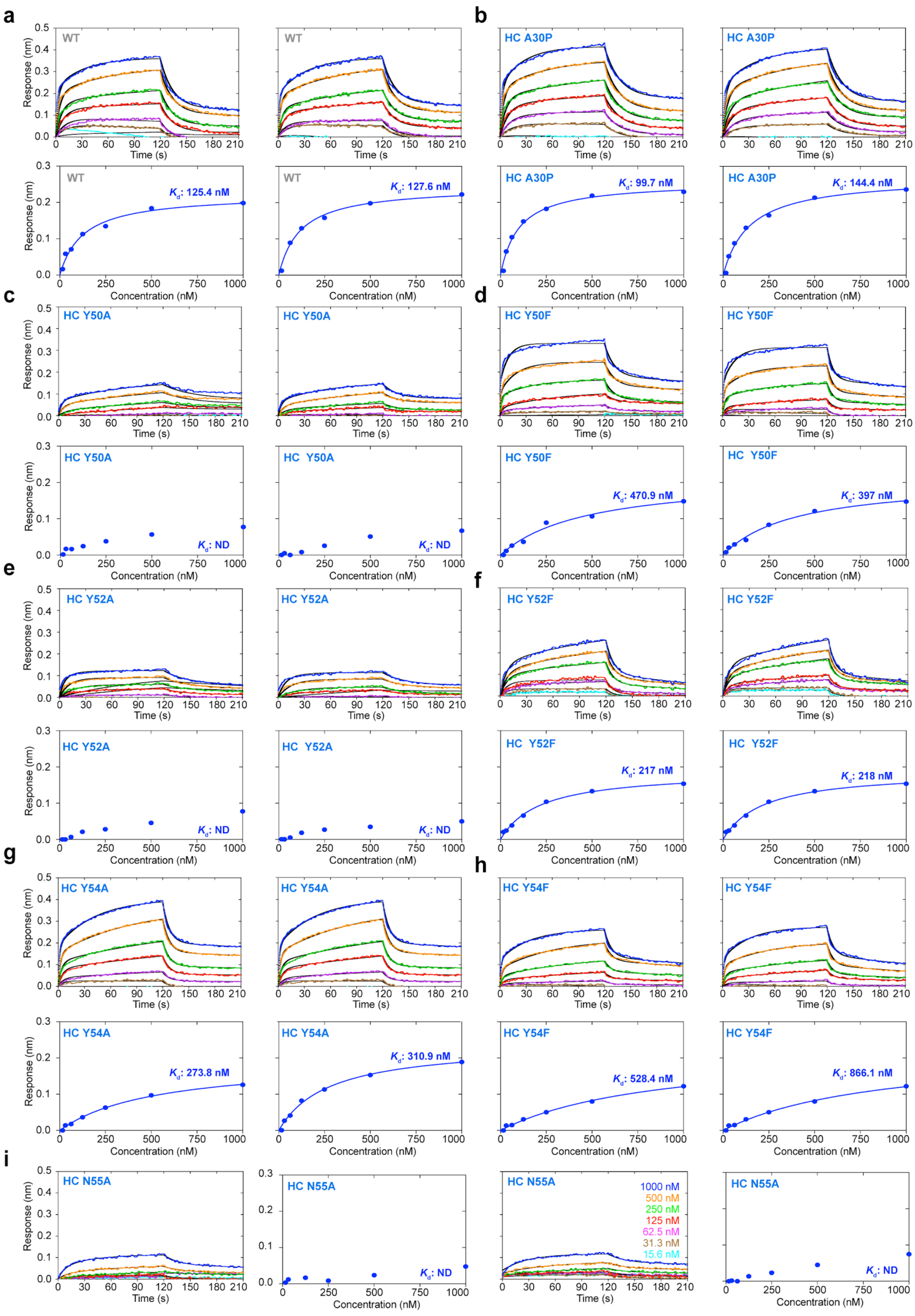

a
b
c
d
e
f
g
h
i
WT
HC A30P
HC Y50A
HC Y50F
HC Y52A
HC Y52F
HC Y54A
HC Y54F
HC N55A
Response (nm)
Time (s)
Concentration (nM)
Kd: 125.4 nM
Kd: 127.6 nM
Kd: 99.7 nM
Kd: 144.4 nM
Kd: ND
Kd: 470.9 nM
Kd: 397 nM
Kd: 217 nM
Kd: 218 nM
Kd: 273.8 nM
Kd: 310.9 nM
Kd: 528.4 nM
Kd: 866.1 nM
1000 nM
500 nM
250 nM
125 nM
62.5 nM
31.3 nM
15.6 nM

**Supplementary Figure 7. Mutational analyses of the J2-dsRNA interface, part 1. a-i**, Additional BLI sensorgrams (upper or left) and steady-state analyses (lower or right) of WT and mutant J2 IgG binding to a 20-bp dsRNA shown in Fig. 1e, at 100 mM KCl

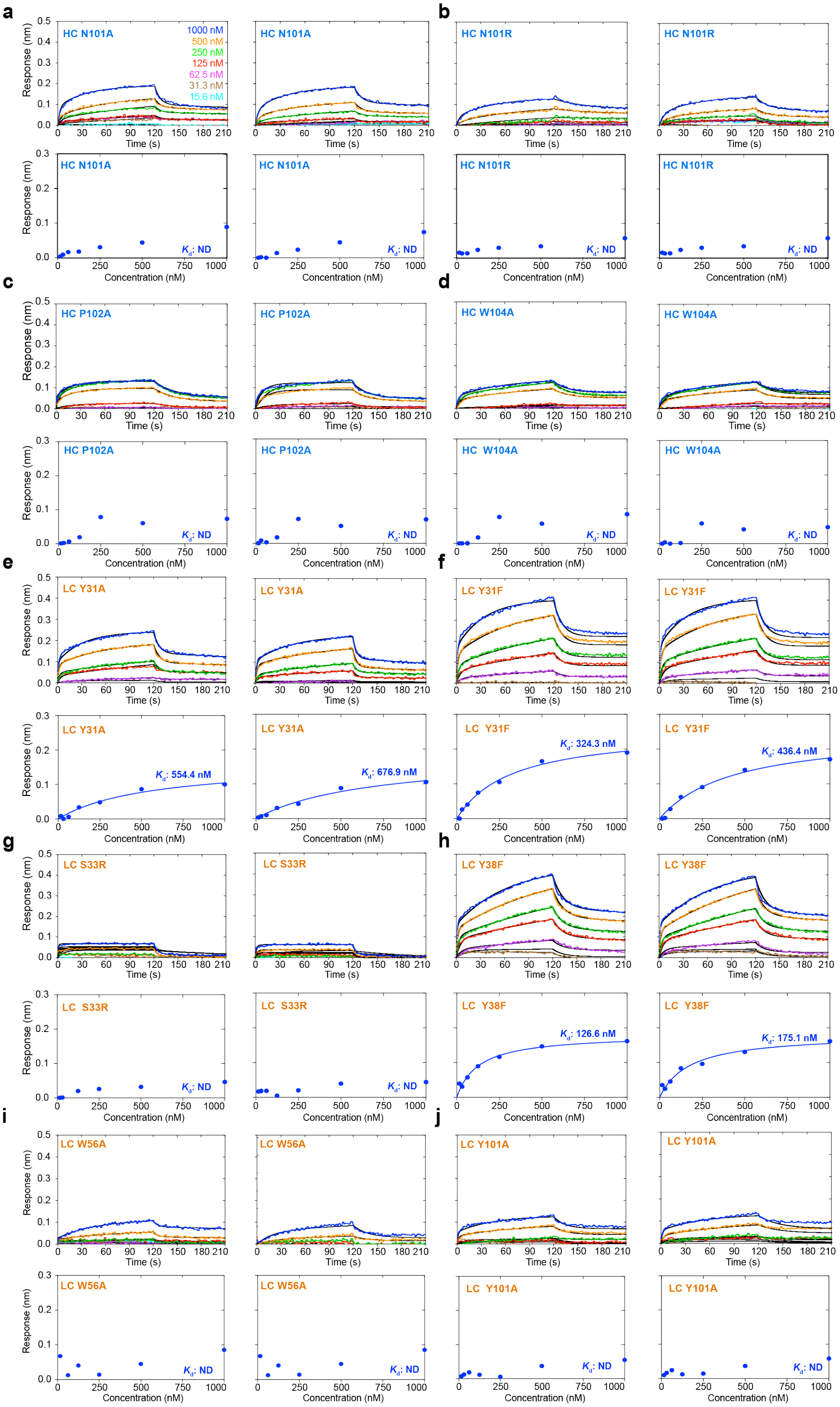
a
b
c
d
e
f
g
h
i
j
HC N101A
HC N101R
HC P102A
HC W104A
LC Y31A
LC Y31F
LC S33R
LC Y38F
LC W56A
LC Y101A
1000 nM
500 nM
250 nM
125 nM
62.5 nM
31.3 nM
15.6 nM
Response (nm)
Time (s)
Concentration (nM)
$K_d$: ND
$K_d$: 554.4 nM
$K_d$: 676.9 nM
$K_d$: 324.3 nM
$K_d$: 436.4 nM
$K_d$: 126.6 nM
$K_d$: 175.1 nM

**Supplementary Figure 8. Mutational analyses of the J2-dsRNA interface, part 2. a-j**, Additional BLI sensorgrams (upper) and steady-state analyses (lower) of WT and mutant J2 IgG binding to a 20-bp dsRNA shown in Fig. 1e, at 100 mM KCl.

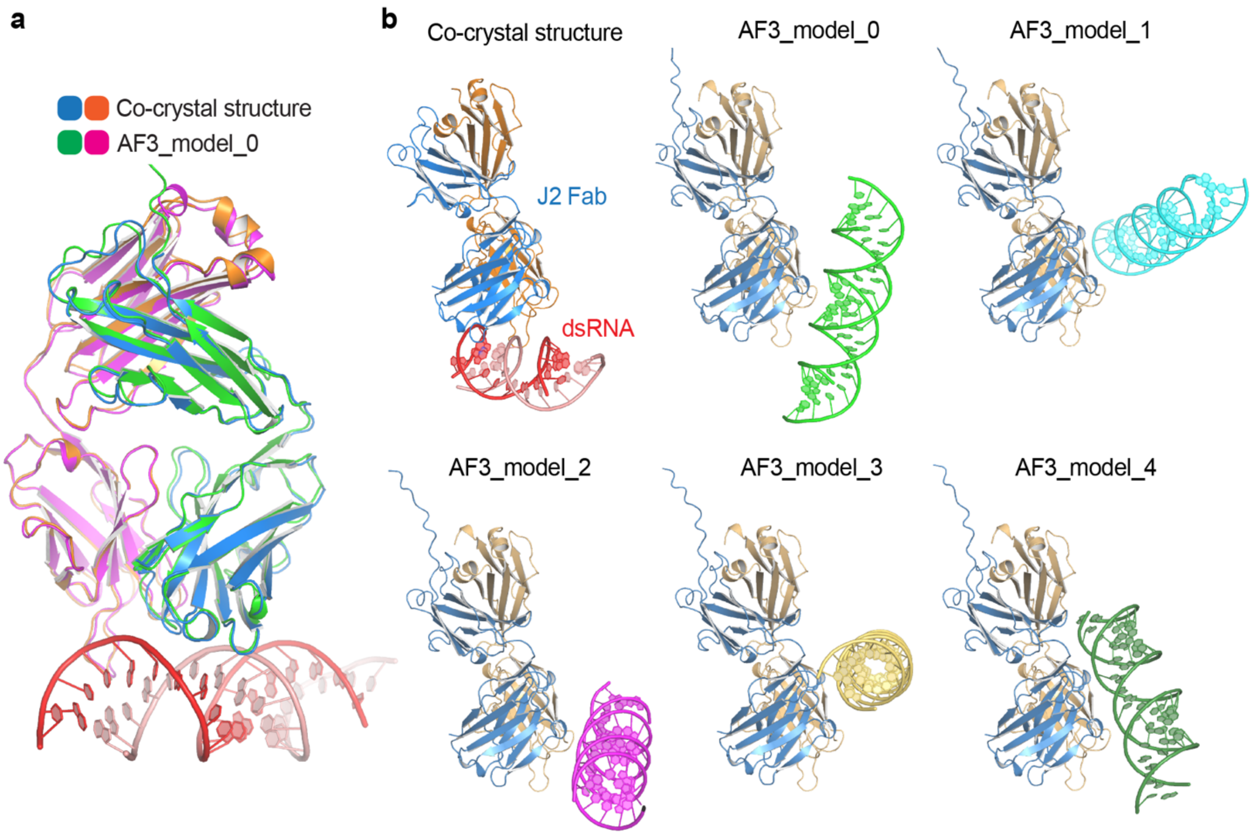


**Supplementary Figure 9. Comparison with AlphaFold 3 predictions of J2 Fab-dsRNA structure. a**, Overlay of J2 Fab-dsRNA co-crystal structure (blue and orange) with the top-scored AlphaFold 3 model. All-atom RMSDs are 0.9-1.0 Å between the proteins. The dsRNA from AlphaFold 3 is omitted here but shown in **b** (top row, middle, green). **b**, Comparison of five top-scored AlphaFold 3-predicted J2-dsRNA complex structural models with the co-crystal structure.

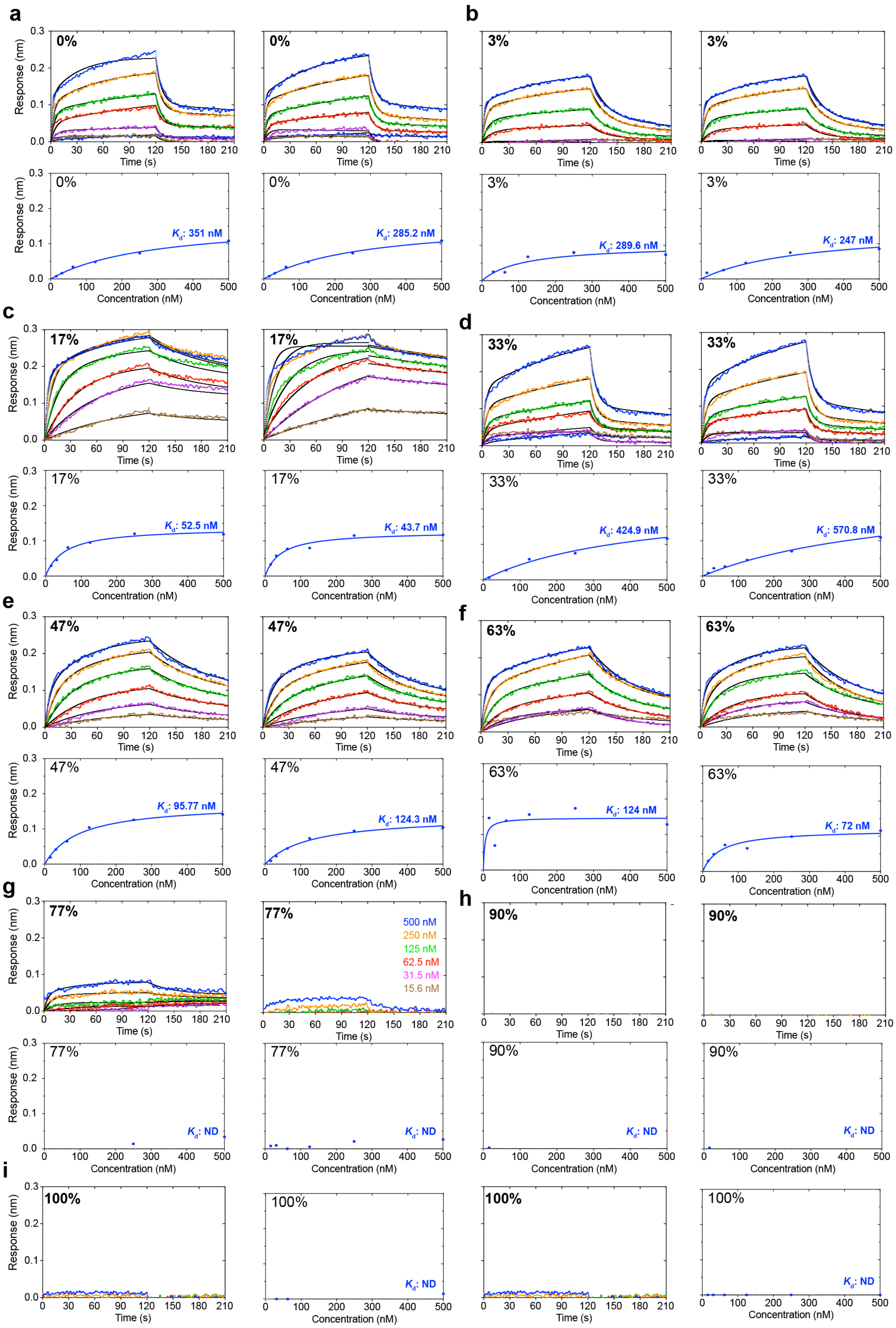

a
0%
0%
$K_d$: 351 nM
$K_d$: 285.2 nM
b
3%
3%
$K_d$: 289.6 nM
$K_d$: 247 nM
c
17%
17%
$K_d$: 52.5 nM
$K_d$: 43.7 nM
d
33%
33%
$K_d$: 424.9 nM
$K_d$: 570.8 nM
e
47%
47%
$K_d$: 95.77 nM
$K_d$: 124.3 nM
f
63%
63%
$K_d$: 124 nM
$K_d$: 72 nM
g
77%
77%
500 nM
250 nM
125 nM
62.5 nM
31.5 nM
15.6 nM
$K_d$: ND
$K_d$: ND
h
90%
90%
$K_d$: ND
$K_d$: ND
i
100%
100%
100%
100%
$K_d$: ND
$K_d$: ND
Response (nm)
Time (s)
Concentration (nM)

**Supplementary Figure 10. Effects of GC contents on J2 IgG binding. a-i**, Additional BLI sensorgrams (upper or left) and steady-state analyses (lower or right) of J2 binding to 30-bp dsRNAs shown in Fig. 4a, with GC contents of 0% (**a**), 3% (**b**), 17% (**c**), 33% (**d**), 47% (**e**), 63% (**f**), 77% (**g**), 90% (**h**), or 100% (**i**), at 200 mM KCl.

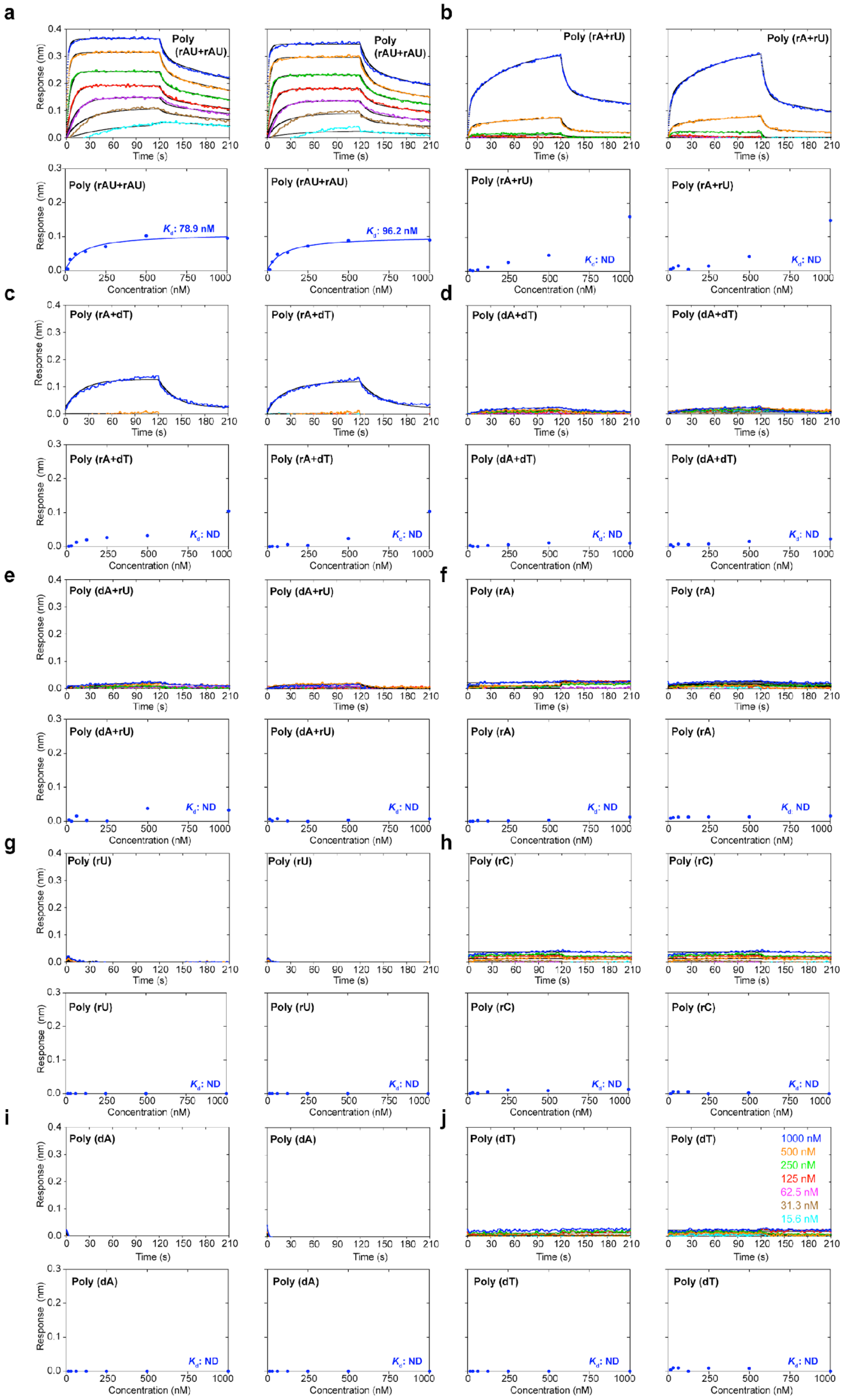
a
b
c
d
e
f
g
h
i
j
Poly (rAU+rAU)
Poly (rA+rU)
Poly (rA+dT)
Poly (dA+dT)
Poly (dA+rU)
Poly (rA)
Poly (rU)
Poly (rC)
Poly (dA)
Poly (dT)
$K_d$: 78.9 nM
$K_d$: 96.2 nM
$K_d$: ND
Response (nm)
Time (s)
Concentration (nM)
1000 nM
500 nM
250 nM
125 nM
62.5 nM
31.3 nM
15.6 nM

**Supplementary Figure 11. J2 IgG interaction with homotypic repeat nucleic acid sequences.** **a-j**, Additional BLI sensorgrams (upper) and steady-state analyses (lower) of J2 binding to repetitive nucleic acid sequences shown in Fig. 5a, at 400 mM KCl.

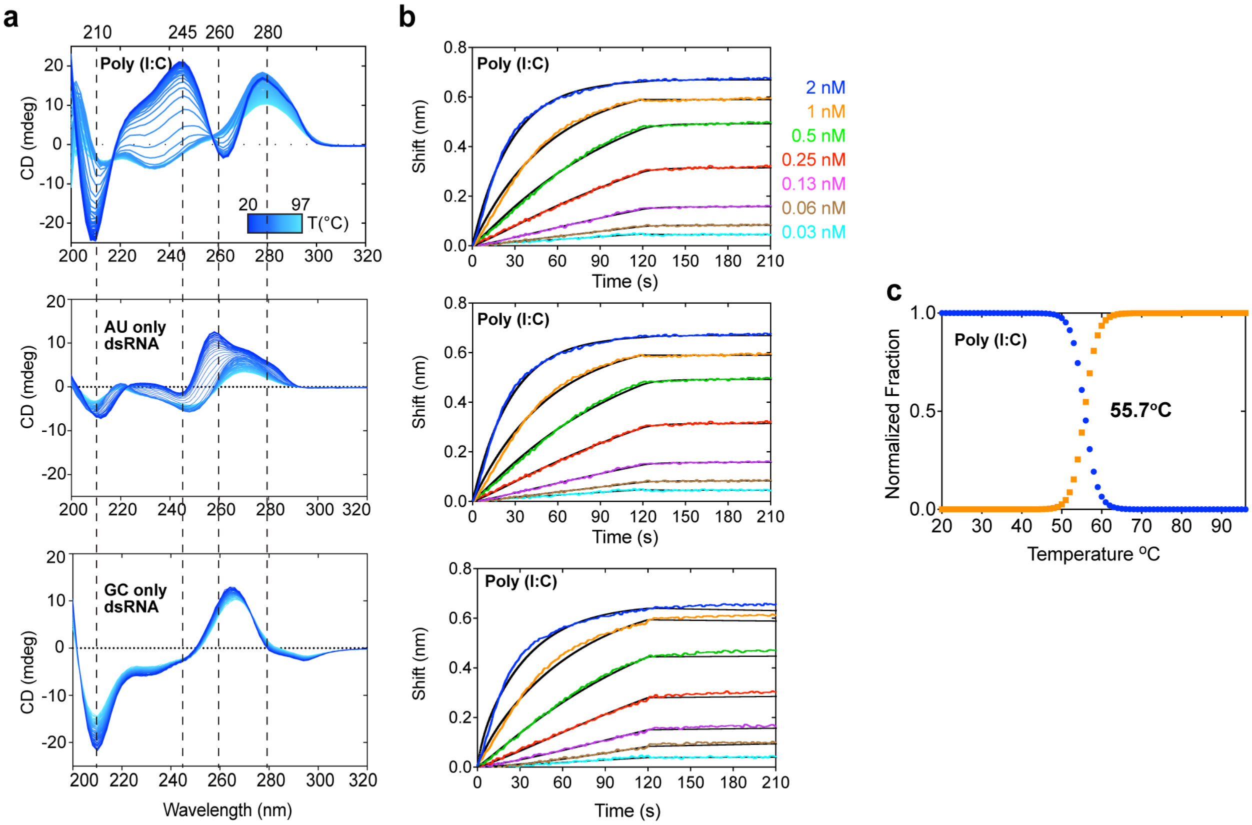


**Supplementary Figure 12. Recognition of poly(rI)-poly(rC) by J2 IgG. a**, Comparison of temperature-scanning CD spectra of poly(rI)-poly(rC) (upper) with 30-bp AU-only (middle) or 30-bp GC-only (lower) dsRNAs in Fig. 4a. **b**, Replicate BLI sensorgrams (at 400 mM KCl) of poly(rI)-poly(rC) binding to J2 IgG. Note the lack of measurable dissociation. $K_d$s could not be reliably determined. **c**, Unfolding transitions of poly(rI)-poly(rC) and $T_m$ derived from data in **a**.

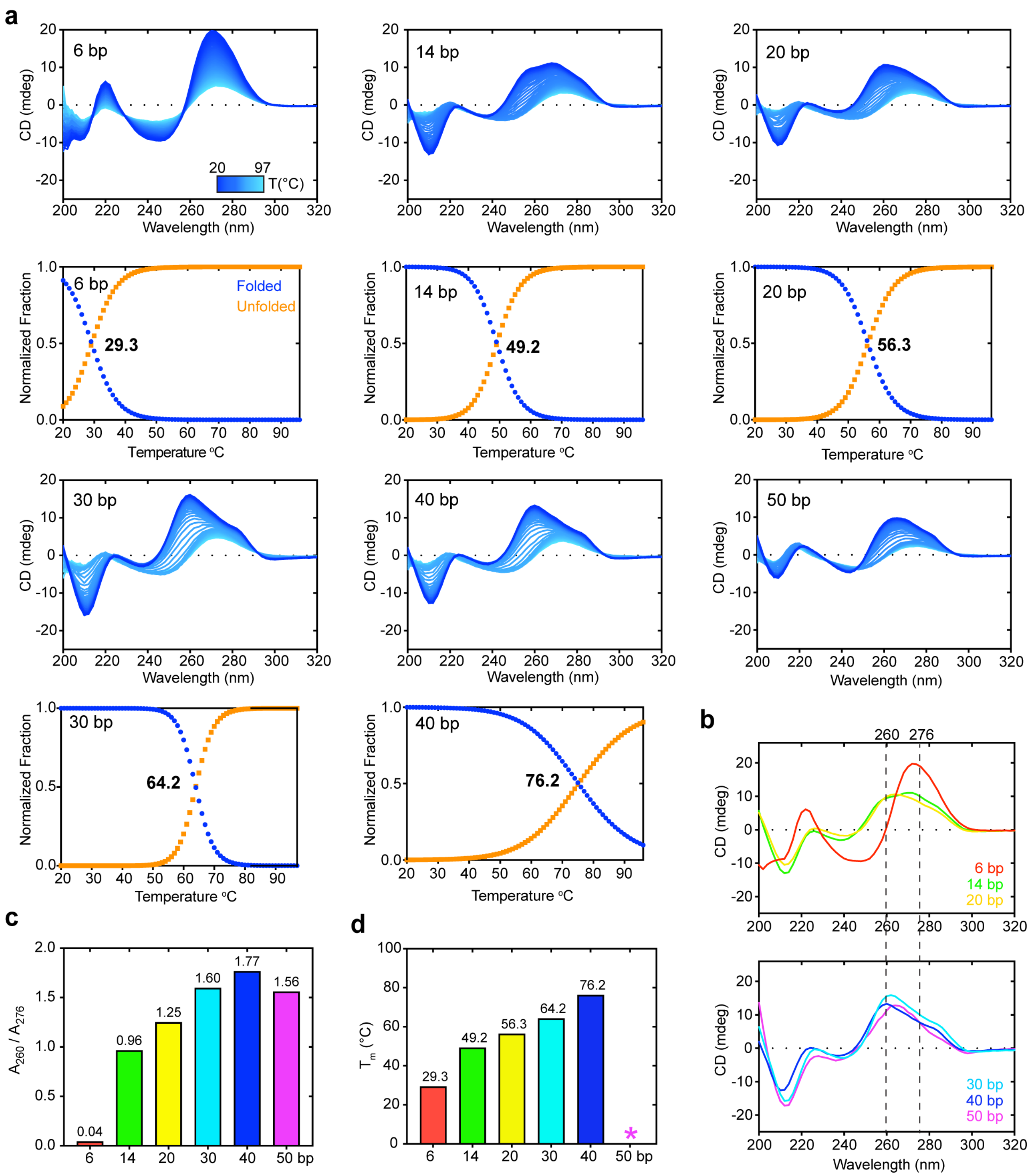


**Supplementary Figure 13. Effects of dsRNA length on RNA helical geometry. a**, Temperature-scanning CD spectra (upper) and unfolding transitions (lower) of 6, 14, 20, 30, 40, and 50-bp dsRNAs in Fig. 1e. **b**, Comparison of the CD spectra in **a** at 20 °C, shown in two groups for clarity. Note the shifting peaks near ~ 260 and 276 nm (marked by vertical dashed lines). **c**, Ratios of CD signals at 260 nm over at 276 nm in **b**. **d**, Melting temperatures ($T_m$s) derived from temperature-scanning CD data in **a**. Asterisk: $T_m$ > 85°C and out of measurable range.

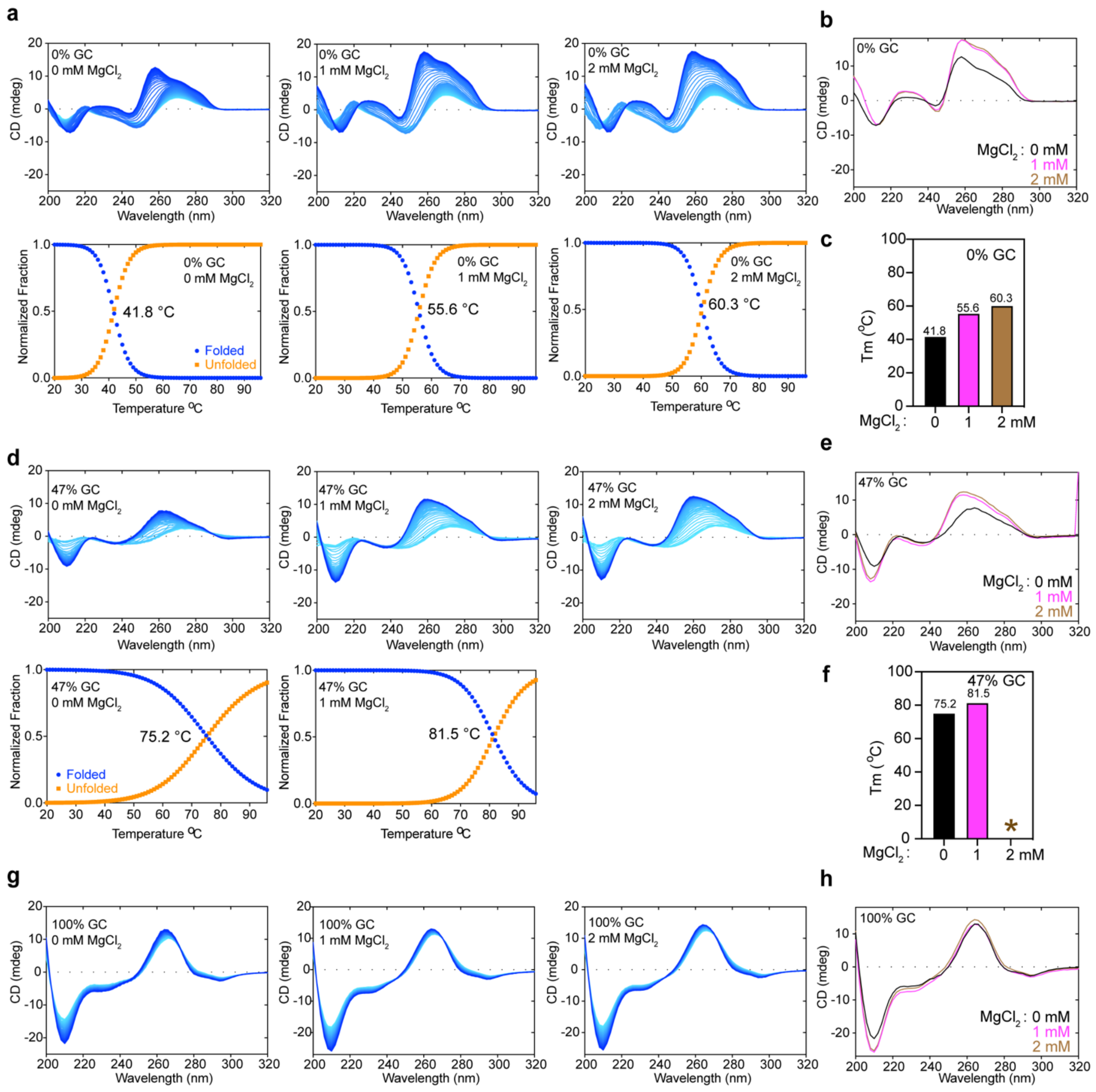


**Supplementary Figure 14. Effects of $Mg^{2+}$ on dsRNA helical geometry.** **a**, Temperature-scanning CD spectra (upper) and unfolding transitions (lower) of 30-bp 0% GC dsRNA in 0 (left), 1 (middle), or 2 (right) mM $Mg^{2+}$. **b**, Comparison of the CD spectra in **a** at 20 °C. **c**, Melting temperatures ($T_m$s) derived from temperature-scanning CD data in **a**. **d**, Temperature-scanning CD spectra (upper) and unfolding transitions (lower) of 30-bp 47% GC dsRNA in 0 (left), 1 (middle), or 2 (right) mM $Mg^{2+}$. **e**, Comparison of the CD spectra in **d** at 20 °C. **f**, $T_m$s derived from CD data in **d**. **g**, Temperature-scanning CD spectra of 30-bp 100% GC dsRNA in 0 (left), 1 (middle), or 2 (right) mM $Mg^{2+}$. **h**, Comparison of the CD spectra in **g** at 20 °C. Asterisk: $T_m$ > 85°C and out of measurable range.
