# Supplementary material for: Structural basis of double-stranded RNA recognition by the J2 monoclonal antibody
Source: Nat Commun. 2025 Dec 13;17:635. doi: 10.1038/s41467-025-67414-z (PMC12816604; doi:10.1038/s41467-025-67414-z)
Supplement: Supplementary file 2 — Reporting Summary [file 41467_2025_67414_MOESM2_ESM.pdf]

nature research

Corresponding author(s): Jinwei Zhang

Last updated by author(s): Nov 17, 2025

Reporting Summary

Nature Research wishes to improve the reproducibility of the work that we publish. This form provides structure for consistency and transparency in reporting. For further information on Nature Research policies, see Authors & Referees and the Editorial Policy Checklist.

Statistics

For all statistical analyses, confirm that the following items are present in the figure legend, table legend, main text, or Methods section.

n/a	Confirmed	
☐	☒	The exact sample size (n) for each experimental group/condition, given as a discrete number and unit of measurement
☐	☒	A statement on whether measurements were taken from distinct samples or whether the same sample was measured repeatedly
☒	☐	The statistical test(s) used AND whether they are one- or two-sided<br>*Only common tests should be described solely by name; describe more complex techniques in the Methods section.*
☒	☐	A description of all covariates tested
☒	☐	A description of any assumptions or corrections, such as tests of normality and adjustment for multiple comparisons
☐	☒	A full description of the statistical parameters including central tendency (e.g. means) or other basic estimates (e.g. regression coefficient) AND variation (e.g. standard deviation) or associated estimates of uncertainty (e.g. confidence intervals)
☒	☐	For null hypothesis testing, the test statistic (e.g. F, t, r) with confidence intervals, effect sizes, degrees of freedom and P value noted<br>*Give P values as exact values whenever suitable.*
☒	☐	For Bayesian analysis, information on the choice of priors and Markov chain Monte Carlo settings
☒	☐	For hierarchical and complex designs, identification of the appropriate level for tests and full reporting of outcomes
☒	☐	Estimates of effect sizes (e.g. Cohen's d, Pearson's r), indicating how they were calculated

Our web collection on statistics for biologists contains articles on many of the points above.

Software and code

Policy information about availability of computer code

Data collection	In-house data collection software was provided by SER-CAT beamline ID-22 at the Advanced Photon Source (APS), Argonne National Laboratory (ANL).
Data analysis	The X-ray diffraction data were indexed, integrated, and scaled by DIALS, version 2.2.5 Molecular replacement phasing was performed using Phenix Phaser, version 1.14. Model building was performed using Coot, version 0.9. Refinement was performed using Phenix.Refine version 1.14. Binding measurements were plotted and analyzed in Prism version 10. CD measurements were analyzed using Global version 3. BLI data were analyzed using GatorOne software. No custom algorithms or softwares were used.

For manuscripts utilizing custom algorithms or software that are central to the research but not yet described in published literature, software must be made available to editors/reviewers. We strongly encourage code deposition in a community repository (e.g. GitHub). See the Nature Research guidelines for submitting code & software for further information.

Data

Policy information about availability of data

All manuscripts must include a data availability statement. This statement should provide the following information, where applicable:

- Accession codes, unique identifiers, or web links for publicly available datasets
- A list of figures that have associated raw data
- A description of any restrictions on data availability

Atomic coordinates and structure factor amplitudes for the J2 Fab in complex with a dsRNA have been deposited at the Protein Data Bank under accession code 9OJV [http://doi.org/10.2210/pdb9OJV/pdb]. Other atomic coordinates used include S9.6 Fab (PDB: 7TQB [http://doi.org/10.2210/pdb7TQB/pdb]). Source data are provided in this paper.

Field-specific reporting

Please select the one below that is the best fit for your research. If you are not sure, read the appropriate sections before making your selection.

☒ Life sciences ☐ Behavioural & social sciences ☐ Ecological, evolutionary & environmental sciences

For a reference copy of the document with all sections, see nature.com/documents/nr-reporting-summary-flat.pdf

Life sciences study design

All studies must disclose on these points even when the disclosure is negative.

Sample size	One crystal sample per X-ray dataset; In vitro assays were performed in biologically independent replicates using distinct samples. Samples sizes are indicated in figures as individual points.
Data exclusions	No data were excluded from the analysis.
Replication	All independent biological replications were successful and included.
Randomization	n/a this study did not use any organisms or participants.
Blinding	n/a this study did not use any organisms or participants.

Reporting for specific materials, systems and methods

We require information from authors about some types of materials, experimental systems and methods used in many studies. Here, indicate whether each material, system or method listed is relevant to your study. If you are not sure if a list item applies to your research, read the appropriate section before selecting a response.

Materials & experimental systems

n/a	Involved in the study	
☐	☒	Antibodies
☐	☒	Eukaryotic cell lines
☒	☐	Palaeontology
☒	☐	Animals and other organisms
☒	☐	Human research participants
☒	☐	Clinical data

Methods

n/a	Involved in the study	
☒	☐	ChIP-seq
☒	☐	Flow cytometry
☒	☐	MRI-based neuroimaging

Antibodies

Antibodies used

J2 antibody produced in house were referenced by and compared to Anti-dsRNA monoclonal antibody J2, Jena Bioscience, Cat. No. RNT-SCI-10010200.

Validation

Validation: Antibodies were validated by vendors and were previously used in published studies.

Validation by vendors:
Purity/Identity: Reducing and Non-reducing SDS-PAGE
Activity: AN-ELISA (relative activity compared to reference J2)

Supplier website: Anti-dsRNA monoclonal antibody J2 (Jena Bioscience, Cat. No. RNT-SCI-10010200):
https://www.jenabioscience.com/images/PDF/RNT-SCI-10010.0003.pdf#page=1.63

References:

Schönborn et al. (1991) Monoclonal antibodies to double-stranded RNA as
probes of RNA structure in crude nucleic acid extracts. Nucleic Acids Res.19:
2993.
Lukacs (1994) Detection of virus infection in plants and differentiation between
coexisting viruses by monoclonal antibodies to double-stranded RNA. J. Virol.
Methods47: 255.
Lukacs (1997) Detection of sense:antisense duplexes by structure-specific
anti-RNA antibodies. In: Antisense Technology. A Practical Approach, C.
Lichtenstein and W. Nellen (eds), pp. 281-295. IRL Press, Oxford

Eukaryotic cell lines

Policy information about cell lines

Cell line source(s)	The Expi-CHO Expression System kit (Expi-CHO-s, ThermoFisher Scientific, Waltham, MA).
Authentication	Cell lines were purchased commercially and were not further validated.
Mycoplasma contamination	ExpiCHO-S (Thermo, A29127) have tested negative for mycoplasma contamination.
Commonly misidentified lines (See ICLAC register)	no commonly misidentified cell lines were used in the study
